# Proteomic Analysis of Tendon Extracellular Matrix Reveals Disease Stage-specific Fragmentation and Differential Cleavage of COMP (Cartilage Oligomeric Matrix Protein)*

**DOI:** 10.1074/jbc.M113.511972

**Published:** 2014-01-07

**Authors:** Stephanie Georgina Dakin, Roger Kenneth Whealands Smith, Dick Heinegård, Patrik Önnerfjord, Areej Khabut, Jayesh Dudhia

**Affiliations:** From the ‡ Department of Clinical Sciences and Services, Royal Veterinary College, Hawkshead Lane, North Mymms, Hatfield, Hertfordshire AL9 7TA, United Kingdom and; § Department of Clinical Sciences Lund, Lund University, BMC Plan C12, SE-22184 Lund, Sweden

**Keywords:** Extracellular Matrix, Inflammation, Mass Spectrometry (MS), Peptides, Proteomics, Tendon

## Abstract

During inflammatory processes the extracellular matrix (ECM) is extensively remodeled, and many of the constituent components are released as proteolytically cleaved fragments. These degradative processes are better documented for inflammatory joint diseases than tendinopathy even though the pathogenesis has many similarities. The aims of this study were to investigate the proteomic composition of injured tendons during early and late disease stages to identify disease-specific cleavage patterns of the ECM protein cartilage oligomeric matrix protein (COMP). In addition to characterizing fragments released in naturally occurring disease, we hypothesized that stimulation of tendon explants with proinflammatory mediators *in vitro* would induce fragments of COMP analogous to natural disease. Therefore, normal tendon explants were stimulated with IL-1β and prostaglandin E_2_, and their effects on the release of COMP and its cleavage patterns were characterized. Analyses of injured tendons identified an altered proteomic composition of the ECM at all stages post injury, showing protein fragments that were specific to disease stage. IL-1β enhanced the proteolytic cleavage and release of COMP from tendon explants, whereas PGE_2_ had no catabolic effect. Of the cleavage fragments identified in early stage tendon disease, two fragments were generated by an IL-1-mediated mechanism. These fragments provide a platform for the development of neo-epitope assays specific to injury stage for tendon disease.

## Teaser

**Background:** Tendon disease is characterized by extensive remodeling of the extracellular matrix.

**Results:** Novel COMP cleavage fragments were identified in both an *in vitro* inflammatory model and natural disease.

**Conclusion:** Inflammatory mediators drive distinct COMP fragmentation at different stages of tendon disease.

**Significance:** Novel COMP neo-terminal fragments provide opportunities for developing markers for tendon injury.

## Introduction

Tendons have an abundant extracellular matrix (ECM)^2^ and are significant causes of morbidity in athletic individuals (1, 2). Repetitive mechanical loading during exercise and inflammation are driving factors in the aetiopathogenesis of injury (3–5). The horse presents an attractive large animal model for the study of the functionally equivalent human Achilles tendon due to the shared characteristics of aging phenotypes (6, 7) and elastic energy-storing function common to the weight-bearing tendons of both species (8, 9). Exercise studies in mature horses have failed to show evidence of an adaptive response to loading, and it is suggested that exercise instead drives degeneration, which precedes clinical injury in adult tendon (10, 11). This concept was supported by studies in the tensional regions of bovine digital flexor tendons where ECM turnover based on mRNA expression was shown to be low (12), and this has been further supported in recent studies in the horse where the remodeling rate of collagen was found to be negligible (half-life of 198 years) (13). Whereas initial studies of human Achilles tendons suggested continued remodeling in adult tendons based on microdialysis (14, 15), recent data has confirmed that the central area of this tendon also has a minimal turnover rate in the adult (16), confirming the horse as a highly relevant model for human tendon disease (17). In contrast, the non-collagenous components of tendon appear to be more rapidly turned over and, therefore, are likely to be influenced by the degradative processes responsible for the hypothesized changes that occur before clinical injury (11, 18).

Equine tendon repair processes are frequently clinically classified into three phases in naturally occurring injury; the acute phase occurs immediately after the initial trauma, lasting only a few days, followed by sub-acute (3–6 weeks) and chronic injury phases (>3 months after injury) (19). This healing response induces profound changes in the composition of the tendon ECM (20–22) associated with the formation of scar tissue that is believed to be responsible for poor functional outcome in both species (23).

COMP is a pentameric glycoprotein belonging to the thrombospondin family (24) that is found in many mechanically loaded tissues including tendon (25). Its functions are thought to include stabilizing the collagen fiber network and catalyzing fibrillogenesis (26, 27) and in assembly, organization, and maintenance of the ECM (28). These roles would explain its strong relationship to tendon mechanical properties in equine tendons (29). COMP levels and fragments are elevated in joint disease and reported in the synovial fluids and serum of patients with rheumatoid arthritis and osteoarthritis (26, 30). Equids with intrathecal digital flexor tendon tears also show elevated COMP levels in tendon sheath fluids (31, 32). COMP degradation is mediated in part by matrix metalloproteinases (MMPs) (33), although MMP-independent pathways involving the aggrecanase ADAMTS-4 (28) also occur.

A growing body of recent evidence from studying tendon tissues from humans (34, 35), equids (3, 36), rodents (37), and *in vitro* models (38) support the role of inflammation in tendinopathy, implicating proinflammatory mediators such as IL-1 and PGE_2_ in disease development and progression. The role of inflammatory cytokines in non-collagenous matrix breakdown has been investigated extensively in cartilage *in vitro* and *in vivo* as typified by loss of COMP and proteoglycans (28, 39–42). Whereas tendon and tendon fibroblasts produce and respond to cytokine stimulation (43), their role in the specific cleavage of ECM proteins is less well documented (28, 39, 40). The ability to detect specific proteolytic cleavage sites is necessary to understand tendon ECM degradative mechanisms that are disease stage-specific for both targeted therapeutic interventions as well as to identify neo-terminal peptide fragments for developing markers for sub-clinical disease for preventative strategies (41). Equine tendons present a more readily attainable source than the human counterpart, permitting targeted investigation of disease through each injury phase as well as normal (uninjured) tendons of a wide age range. The aim of this study was to identify COMP fragments generated at different stages of tendon disease and to relate these to those induced specifically by IL-1β and PGE_2_ *in vitro*. This is the first comprehensive analysis of tendon ECM degradation in acute and chronic disease, and we identify novel COMP fragments in natural disease that are produced by an IL-1β-driven mechanism.

## EXPERIMENTAL PROCEDURES

### 

#### 

##### Collection of Equine Tendons

Equine forelimbs from Thoroughbred or Thoroughbred cross-breed horses aged between 2 and 20 years were obtained from an abattoir or local equine referral hospital with known history of injury and the tensile (mid-metacarpal) region of the superficial digital flexor tendon (SDFT) harvested within 4 h of death. Tendons were grouped as sub-acutely injured (3–6 weeks post injury, *n* = 6, mean age 9 ± 5 years) or chronically injured (>3 months post injury, *n* = 9, mean age 13 ± 4 years) as described before (3). Tendon injuries were aged based on historical information obtained from either the owner or referring veterinary surgeon before euthanasia of the horse. Tendons were classified as normal based on their macroscopic postmortem appearance, which included lack of visible signs of swelling of the tendon body and a consistent pattern of fascicles on hematoxylin- and eosin-stained sections (*n* = 19, mean age 8 ± 5 years).

##### Preparation of Tendon Explants for Tissue Culture

Macroscopically normal tendons were used for *in vitro* experiments and derived from horses (*n* = 10) between 7 and 14 years of age (mean 10 ± 3 years). SDFTs were aseptically dissected from the limb, and after removal of the paratenon, tendon explants were cut in a tissue culture flow hood using three parallel sterile microtome blades (Surgipath) inserted into a custom-designed cutting template to create 2 × 2 × 37-mm pieces along the longitudinal axis of the tendon (6). Two explants per well were cultured in serum-depleted DMEM (3 ml per well) containing 5% penicillin and streptomycin (Invitrogen) in tissue culture 6-well plates (VWR) at 37 °C in humidified atmosphere (5% CO_2_ and air). This method of preparing the explant tissue consistently produced average wet weights of 300 mg (±30 mg).

To assess the effects of proinflammatory mediators on release of tendon matrix components, explants were stimulated with human recombinant IL-1β (5 ng·ml^−1^) (Calbiochem) or PGE_2_ (0.01 or 1.0 μm) (Sigma), and release of total collagen and COMP into tissue culture media was quantified and compared with non-stimulated controls. After cutting (time 0), explants were incubated in serum-depleted media and rested for 24 h to allow the tissue to adapt to the culture environment. Twenty-four hours after explant cutting, media were replaced, and samples were stimulated with proinflammatory mediators. The following inhibitors were added to the experimental system to identify inflammation-relevant release of COMP by intervention of the PGE_2_ synthesis pathways (including PGE_2_ synthesis via IL-1β): 1.0 μm Firocoxib (Merial, France), 20 μm Ilomastat (Calbiochem), 400 ng·ml^−1^ recombinant equine IL-1Ra (R&D Systems). Media were harvested and analyzed at 48, 72, and 96 h (post-cutting) with complete media replacement at each interval.

##### Viability of Tendon Explants in Culture

To demonstrate viability of tendon cells at the measured experimental time points, live-dead staining was performed with 4 μm ethidium bromide and 2 μm calcein AM (Sigma) in PBS containing 5.6 mm glucose, 0.5 mm MgCl_2_, and 0.9 mm CaCl_2_ for 1 h in dark conditions before confocal microscopy (Leica Microsystems, Milton Keynes, UK). Viability of explants cultured in serum deplete DMEM containing 5% penicillin and streptomycin were compared at 24 and 120 h after cutting, with explants incubated in 2% sodium azide for 24 h as a negative control. ImageJ software (NIH Version 1.42) was used to ascertain the proportion of live and dead cells.

##### Sircol Collagen Assay

The Sircol collagen assay (Biocolor Ltd) was used to quantify release of triple helical collagens into tissue culture media as per the manufacturer's instructions. Briefly, 200 μl of culture media was assayed in triplicate in 96-well microtiter plates, and the final absorbance was read at 555 nm (Sunrise micro plate reader, Tecan, Männedorf, Switzerland). The substrate background absorbance values were subtracted from absorbance readings, and a standard curve was generated using bovine type I collagen as specified by the assay manufacturer. Results were adjusted to represent collagen release per mg of explant wet weight measured at the termination of the experiment.

##### COMP ELISA

The COMP ELISA was an in-house assay that has been used successfully with equine samples, the methodology for which is described in detail elsewhere (31, 32). COMP release was determined in samples of tissue culture media incubating tendon explants under differing experimental conditions. Results were expressed as μg/ml and subsequently adjusted to represent COMP release per mg of explant wet weight.

##### SDS/PAGE and Western Blotting of COMP

Western blotting of samples of culture media was used to compare the effects of proinflammatory mediators on the release of COMP from tendon. Western blotting of undiluted media was performed under reduced and non-reduced conditions. Samples were reduced by the addition of dithiothreitol to 0.1 m and heated to 95 °C for 5 min before electrophoresis on 8–10% SDS/PAGE gels. After electrophoresis, proteins were transferred for Western blotting (Bio-Rad) onto PVDF membranes (GE Healthcare). Membranes were blocked overnight in Tris-buffered saline (0.02 m Tris-base, 0.02 m Tris HCl, and 0.05 m NaCl) in 1% Triton (TBST buffer) containing 8% powdered skimmed milk (Marvel) and 2% bovine serum albumin (Sigma). After washing in TBST (3 times for 10 min each), membranes were incubated with the COMP primary antibody (25) in a buffer containing 4% (w/v) milk and 1% (w/v) BSA in TBST at a 1:1000 dilution for 2 h. Membranes were washed 3 times as before and incubated with anti-rabbit IgG HRP-linked secondary antibody (Cell Signaling Technology®) in antibody buffer for 2 h at a 1:2000 dilution. Antibody-positive protein bands were visualized using enhanced chemiluminescence (ECL) reagent and film (GE Healthcare). Densitometric analysis of protein bands on non-reduced blots was performed using ImageJ software (NIH Version 1.42) using sequential exposures of films to avoid saturation artifacts.

##### Proteomic Analyses Using Mass Spectrometry

Liquid chromatography mass spectrometry (LC-MS) using a quadruple time-of-flight mass spectrometer (Q-TOF) (Q-TOF micro, Waters) were performed on samples of experimental media and extracts of normal and injured SDFTs. Multiple reaction monitoring (MRM) analyses were performed using another LC-MS system comprising of an Easy nano-LC^TM^ (Thermo Scientific) triple quadrupole instrument (TSQ Vantage^TM^, Thermo Scientific) on media samples from tendon explants *in vitro* for one experiment at 72 h, enabling selective quantification of known peptides. The relative costs associated with the use of MS-MS precluded analyses of large numbers of samples. Proteomic analyses using MS-MS were performed to identify ECM proteins and neo-termini of COMP fragments released into media from stimulated and control normal tendon explants *in vitro*, as COMP was the most abundant ECM protein released from tendon explants in culture. For these proteomic analyses, explants were cut (time 0) and rested for 48 h, as resting for 24 h in pilot studies suggested this was of insufficient duration due to a significant quantity of proteins, and peptides released before base-line levels were reached at 48 h. At 48 h, media were replaced, and the tissue was stimulated with proinflammatory mediators for a further 24 h. All media samples for proteomic analyses were analyzed at the 72 h time point after explant cutting.

##### Preparation of Media Samples for the Q-TOF MS

Care was taken to avoid contamination of samples with skin and hair keratins. 100 μl of media from each sample was reduced with 4 mm dithiothreitol and agitated at 56 °C for 30 min. Samples were alkylated with 16 mm iodoacetamide at room temperature in the dark for 1 h. Samples were digested with 0.5 μg trypsin gold (Promega, Madison, WI) overnight at 37 °C on a shaker for 16 h. Samples were dried in a SpeedVac and suspended in 100 μl of 0.2% formic acid whereof 10 μl were purified and desalted using homemade reversed phase tips, 4 discs thick (47-mm Empore C18 extraction discs, 3M, Minneapolis, MN) as described before (44, 45) and subsequently dried and redissolved in 20 μl 0.2% formic acid before injection onto the Q-TOF MS.

##### Preparation of Media Samples for the Triple Quadrupole MS

Media samples (10 μl of trypsin digest, see above) were cleaned with reversed-phase C18 columns according to the manufacturer's instructions (SUM SS18V); columns were purchased from the Nest Group.

##### Preparation of Tendon Tissue Extracts for QTOF MS Analysis

Proteomic analyses were also performed on tissue extracts from macroscopically normal, sub-acute (3–6 weeks post injury) and chronic injured (>3 months post injury) SDFTs to investigate the effect of injury on matrix protein composition. Samples of normal, sub-acute, and chronic injured SDFTs (*n* = 3 of each) stored at −80 °C were finely diced, and 15 volumes of 4 m guanidine HCl containing protease inhibitors (1:100 dilution of protease inhibitor mixture III, Calbiochem) and 10 mm EDTA were added. Samples were rotated at room temperature for 48 h and then centrifuged at 4 °C for 15 min at 13, 000 × *g* to recover the extract. 50 μl of extract was reduced in 10 mm dithiothreitol at 56 °C on a shaker for 30 min and alkylated at 40 mm iodoacetamide for 60 min at room temperature in the dark. Extracts were precipitated with ice-cold ethanol (9:1) overnight at 4 °C before centrifugation (13,200 × *g* at 4 °C for 30 min) followed by an ethanol wash for 4 h at −20 °C to remove residual guanidine HCl and other salts. Samples were dried in a SpeedVac and suspended in 100 μl of 0.1 m triethylammonium bicarbonate, pH 8.5, before trypsination with 1 μg of trypsin gold at 37 °C on a shaker for about 16 h. Samples were purified and concentrated using homemade reversed phase tips.

##### MS-MS Data Analyses of COMP Peptides

For Q-TOF LC-MS (Q-TOF micro, Waters) mass spectrometric raw data were processed using Protein Lynx 2.1 (Waters). Peptide and neo-termini searches were performed using the databases (SwissProt 56.9 and ENSEMBL) and MASCOT MS/MS Ions Search (version 2.1). Due to the presence of collagens in tendon, hydroxylation of proline residues were allowed in database searches. MASCOT search parameters included carbamidomethylation of cysteine as fixed modification, deamidation (Asn and Gln), and oxidation (Met and Pro) were considered as variable modifications. Other MASCOT search parameters were: monoisotopic masses, ±0.2-Da peptide mass tolerance, ±0.2-Da fragment mass tolerance, max miss cleavage of 2, ion score minimum 20, only highest ranked peptide matches, and taxonomy *Equus caballus*. MRM data were analyzed using the Skyline 1.4 software (MacCoss Lab Software, University of Washington).

##### Statistical Analyses

Statistical analyses were conducted using SPSS PASW Statistics 18 (SPSS Inc.). Linear mixed models were used to analyze COMP release to account for effects of horse, experimental condition, and time. Analyses for release and percentage change in release relative to the respective controls are shown for COMP. *p* < 0.05 was considered statistically significant.

##### Ethics Statement

Ethical approval for the collection of postmortem equine tendons from an abattoir or local equine veterinary referral hospital for this study was sought and approved from the Ethics and Welfare Committee at the Royal Veterinary College (URN 2011 1117).

## RESULTS

### Tendon Explant Viability in Vitro

**FIGURE 1.**
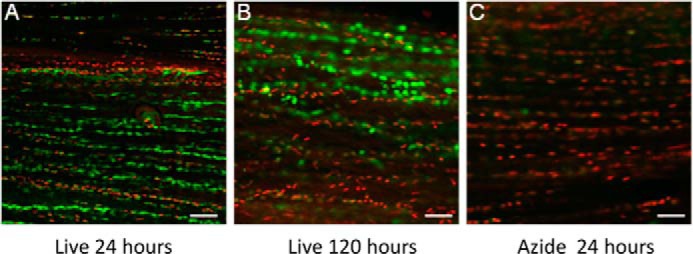
**Viability of tendon fibroblasts in explant culture.** Confocal fluorescence micrographs of tendon sections treated with calcein AM to denote cytoplasmic staining of live cells (*green*) and ethidium bromide for nuclear staining (*red*) of live and dead cells. Explants after 24 h (*A*) and 120 h (*B*) are shown. Cell viability was 90 ± 5% after 24 h in culture and 60 ± 5% after 120 h. *C*, azide-treated explant after 24 h was used as a negative control. All experiments were performed within the 120-h culture period. *Scale bar* = 20 μm.

Confocal images illustrating explant viability are shown in Fig. 1. After culture for 24 h in serum-deplete media, 90 ± 5% of cells within SDFT explants were viable. Explant viability was 60 ± 5% after 120 h in culture. The majority of cell death present was located along the periphery of the cut edges in a linear pattern along rows of tenocytes.

### Effects of IL-1β and PGE_2_ on the Tendon ECM

#### 

##### Minimal Collagen Degradation by IL-1β and PGE_2_ Stimulation

Soluble collagens released from tendon explants treated with IL-1β or PGE_2_ was minimal and ranged between 0.01 and 0.03 μg/mg of tissue in all samples between 24 and 96 h, but this was not significantly different from control cultures.

##### Release of COMP after IL-1β and PGE_2_ Stimulation

**FIGURE 2.**
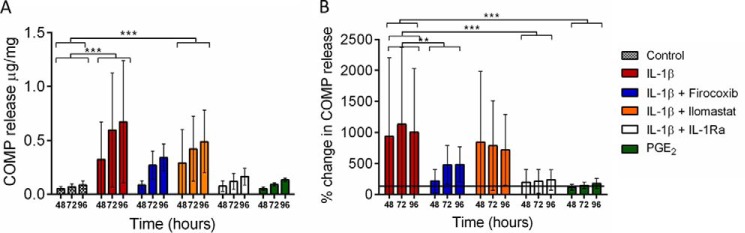
**Release of COMP into media from SDFT explants in culture.** Normal SDFT explants were derived from 3 horses ages between 9 and 13 years (mean 10 ± 2 years). Explants were rested for 24 h after cutting before media replacement and stimulation commencing. Media were harvested at 48, 72, and 96 h with complete replacement at each time point. *A*, mean cumulative COMP release showing significantly increased release with IL-1β stimulation alone and in combination with 20 μm Ilomastat. COMP release after stimulation with 1.0 μm PGE_2_ was similar to non-stimulated controls. *B*, mean percentage change in COMP release relative to the respective control. The addition of either 400 ng·ml^−1^ IL-1Ra or 1.0 μm Firocoxib significantly reduced IL-1β-mediated COMP release. *Error bars* represent S.D. ** *p* < 0.01; *** *p* < 0.001.

Mean COMP levels in media were 0.26 ± 0.1 μg/mg in the first 24-h equilibration period in unstimulated cultures. This 24-h period was not included in statistical analyses. The cumulative release of COMP significantly increased in all samples with times between 48 and 96 h (*p* = 0.008) and was substantially increased by IL-1β (∼10-fold increase) compared with control samples (Fig. 2*A*). Although there was increased COMP release after stimulation with 1.0 μm PGE_2_, this was not statistically significantly different compared with controls. COMP release was significantly reduced by the addition of IL-1Ra (400 ng·ml^−1^) and Firocoxib (1.0 μm) (*p* < 0.001 and *p* = 0.004, respectively) but not by Ilomastat (Fig. 2*B*).

### Analysis of ECM Proteins in Media by Western Blotting

**FIGURE 3.**
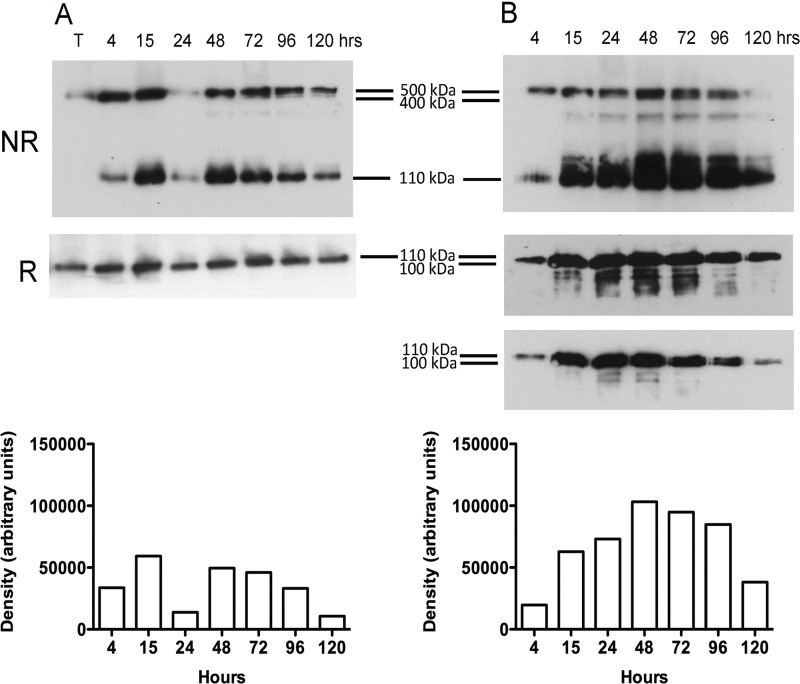
**COMP release into culture media from normal SDFT explants.** Representative Western blots of media from experimental samples showing COMP release with time from control (*A*) and IL-1β stimulated tendon explants (*B*) under non-reduced (*NR*) and reduced (*R*) conditions. Densitometric analysis of protein bands is shown for non-reduced Western blots. COMP release was enhanced by stimulation of explants with IL-1β compared with non-stimulated controls, with maximal release occurring at 48 h and the appearance of a number of distinct peptides <100 kDa. In IL-1β-stimulated explants, monomeric COMP appears as a doublet under reduced conditions from 15 to 120 h after explant cutting, which is better demonstrated in a lower exposure of the blot in a lower panel. *T*, equine SDFT extract (total protein loaded 10 μg) prepared in 4 m (guanidine HCl) was used as a loading standard and as a positive control for COMP, which is present mostly as a 550-kDa pentamer in the tissue.

**FIGURE 4.**
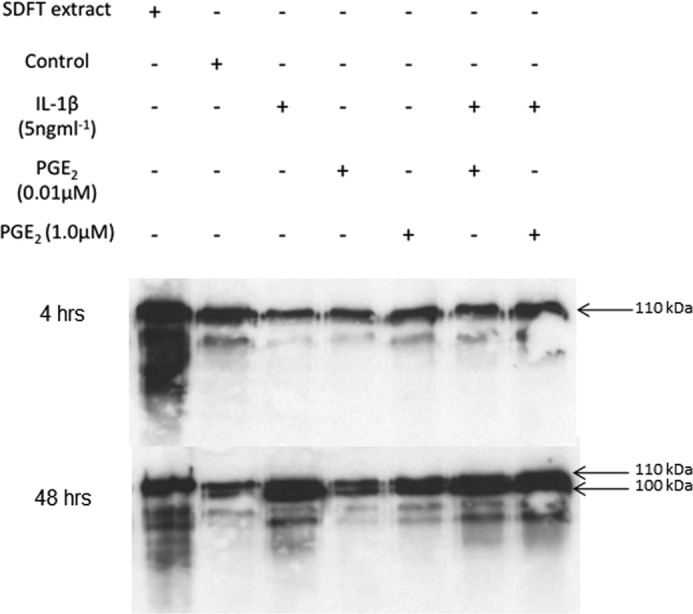
**Differential effects of IL-1β and PGE_2_ on COMP release.** Representative Western blot of media samples harvested at 4 h and 48 h. Tendon extract = positive control (10 μg total protein); control = media from unstimulated tendon at the same respective time point. COMP release and fragmentation were enhanced by stimulation with IL-1β at 48 h with lower molecular weight fragments present including the appearance of the 100-kDa peptide (doublet). The addition of PGE_2_ resulted in the release of both intact and fragmented COMP (100 kDa) at 48 h. Smaller fragments were detected with the higher dose of PGE_2_, but these were present in low abundance compared with stimulation with IL-1β.

Analysis of culture medium from the tendon explant experiments confirmed the release of COMP from the tendon ECM over the 120-h culture period (Fig. 3). COMP was released in a number of known multimeric forms (25) that could be identified in non-reducing conditions (Fig. 3*A*, *NR*) and migrated as a single monomeric form in reducing conditions (Fig. 3*A*, *R*). The release of COMP from the tissue increased to a maximum at 48 h after explant cutting. Stimulation with 5 ng·ml^−1^ IL-1β induced additional release of COMP as early as 15 h but was most marked after 48 h compared with controls (Fig. 3*B*, *R* and *NR*) and included an ∼100-kDa protein fragment not present in the control cultures under reduced conditions (Figs. 3 and 4). Fragments smaller than 100 kDa were observed with IL-1β after 15 h of stimulation (Fig. 3). Qualitative assessment of Western blots loaded with the same volume of media (Fig. 4) supported the increased release of both monomeric (110 kDa) and fragmented COMP (∼100 kDa) with 1.0 μm PGE_2_, which was not significant by ELISA (Fig. 2*A*). However, fragments smaller than 100 kDa were only observed after stimulation at the higher PGE_2_ dose (1.0 μm), and in contrast to IL-1β these fragments were present in relatively low abundance (Fig. 4). Combined addition of IL-1β with low or high doses of PGE_2_ had no additional effect on COMP release.

### Proteomic Analyses of Culture Media by Mass Spectrometry

#### 

##### LC-MS and Q-TOF Analyses

Semiquantitative LC-MS analyses showed COMP to be the most abundant ECM protein released in all media samples from stimulated and non-stimulated explants followed by thrombospondin-4, clusterin, and fibronectin (Table 1). Stimulation with IL-1β (5 ng·ml^−1^) induced an ∼2-fold increase in COMP release compared with other experimental conditions. Consequently further analyses were focused on COMP and its related fragments. A list of neo-terminal peptides of COMP produced under different inflammatory stimuli is shown in Table 2. Five of the COMP neo-terminal peptides present in control samples were also common to samples stimulated with each pro-inflammatory mediator (*i.e.* present in all samples). The addition of IL-1β (5 ng·ml^−1^) generated a greater number of neo-terminal peptides of COMP compared with controls and stimulation with 1.0 μm PGE_2_. The neo-terminal peptides generated from explants stimulated with 0.01 μm PGE_2_ were identical to those released from controls. Interestingly, combined stimulation of tendon explants with IL-1β and low dose (0.01 μm) PGE_2_ generated a greater number of neo-terminal peptides in contrast to IL-1β with high dose (1.0 μm) PGE_2_ (Table 2).

**TABLE 1:** **Top ranking identified tendon ECM proteins (by protein score) released into media by proinflammatory mediators** Protein abundance was measured by semi-quantitative LC-MS and is indicated by the exponentially modified protein abundance index in parentheses (emPAI).

Rank of protein	Control	IL-1β (5 ng·ml^−1^)	PGE_2_ (0.01 μm)	PGE_2_ (1.0 μm)
1	COMP (3.44)	COMP (6.41)	COMP (3.84)	COMP (3.08)
2	Thrombospondin4 (1.35)	Thrombospondin4 (1.7)	Thrombospondin4 (1.28)	Thrombospondin4 (0.92)
3	Fibronectin (0.6)	Clusterin (0.85)	Clusterin (0.85)	Clusterin (0.5)
4	Clusterin (0.5)	Fibronectin (0.51)	Fibronectin (0.74)	Fibronectin (0.49)
5	Decorin (0.09)	Interleukin-6 (0.56)	Collagen3 (0.23)	Decorin (0.3)
6	Thrombospondin1 (0.06)	Collagen3 (0.17)	Thrombospondin1 (0.06)	Collagen3 (0.29)
7	CILP-1 (0.06)	Thrombospondin1 (0.11)	CILP-1 (0.06)	Thrombospondin1 (0.08)
8	Collagen1 (0.06)	Aggrecan (0.05)	Aggrecan (0.03)	Aggrecan (0.02)

**TABLE 2:** **Summary of Q-TOF LC-MS analyses for neo-terminal peptides of COMP released into media from stimulated tendon explants** Numbers indicate the position of the peptide within the equine COMP protein sequence (NCBI accession AF325902).

Control	IL-1β	1.0 μm PGE_2_	0.01 μm PGE_2_ + IL-1β	1.0 μm PGE_2_ + IL-1β
228–238 C↓PDGTPSPCHEK↓A*^a^*	37–48 E↓LQETNAALQDVR↓E	89–108 Q↓CAPGSCFPGVACTQTASGAR↓C	37–48 E↓LQETNAALQDVR↓E	81–88 R↓VSVRPLAQ↓C
294–303 V↓PNSGQEDADR↓D*^a^*	88–108 A↓QCAPGSCFPGVACTQTASGAR↓C		88-108 A↓QCAPGSCFPGVACTQTASGAR↓C	89–108 Q↓CAPGSCFPGVACTQTASGAR↓C
642–649 S↓TGPGEQLR↓N*^a^*	89–108 Q↓CAPGSCFPGVACTQTASGAR↓C		89 – 108 Q↓CAPGSCFPGVACTQTASGAR↓C	
724–736 F↓CFSQENIIWANLR↓Y*^a^*	203–222 F↓QCGPCQPGFVGDQASGCRPR↓A		254–266 C↓AVGWAGNGLLCGR↓D	
726–736 F↓SQENIIWANLR↓Y*^a^*	254 – 266 C↓AVGWAGNGLLCGR↓D		725–736 C↓FSQENIIWANLR↓Y	
	269–279 T↓DLDGFPDEKLR↓C			
	320–330 V↓PNEGDNCPLVR↓N			
	600–613 F↓GYQDSSSFYVVMWK↓Q			

*^a^* Denotes the neo-terminal peptide was present in all samples, including 0.01 μM PGE2-stimulated samples, which were the same as controls. Sequences shown with an arrow represent the cleavage site, and the residue following the arrow represents the neo-terminus. IL-1β dose is 5 ng·ml^−1^ for all samples.

##### Targeted Mass Spectrometry Using MRM

**FIGURE 5.**
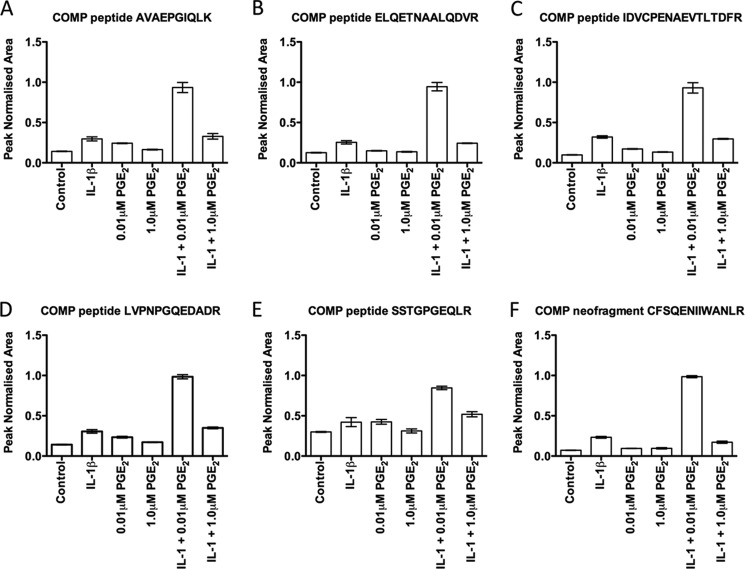
**MRM analyses of COMP peptides released into media after 72 h in culture: AVAEPGIQLK (*A*), ELQETNAALQDVR (*B*), IDVCPENAEVTLTDFR (*C*), LVPNPGQEDADR (*D*), SSTGPGEQLR (*E*), and COMP neo-terminal fragment CFSQENIIWANLR (*F*).** Each sample was run on the MRM in triplicate, and the mean values with S.D. are shown for each experimental condition. MRM analyses were conducted on triplicate samples from one experiment and not biological replicates; hence, statistical analyses were not performed. Peak normalized area represents the summed area of the ion peak transitions for each peptide measured. There was a trend for enhanced COMP peptide/neo-N-terminal fragment release with IL-1β compared with non-stimulated controls. For all peptides, maximal release occurred after stimulation with both IL-1β and 0.01 μm PGE_2_.

MRM analyses were performed on experimental media samples after 72 h in culture from one experiment. Using this approach it was possible to perform relative quantifications of known peptides and neo-terminal fragments of COMP between samples stimulated with proinflammatory mediators and non-stimulated controls. There was a general trend for IL-1β-stimulated tendon explants to release higher quantities of peptides compared with non-stimulated controls (Fig. 5, *A–F*). The greatest numbers of peptides were generated when explants were incubated with both IL-1β and low dose (0.01 μm) PGE_2_.

### Proteomic Analyses of Normal and Injured Tendon

**FIGURE 6.**
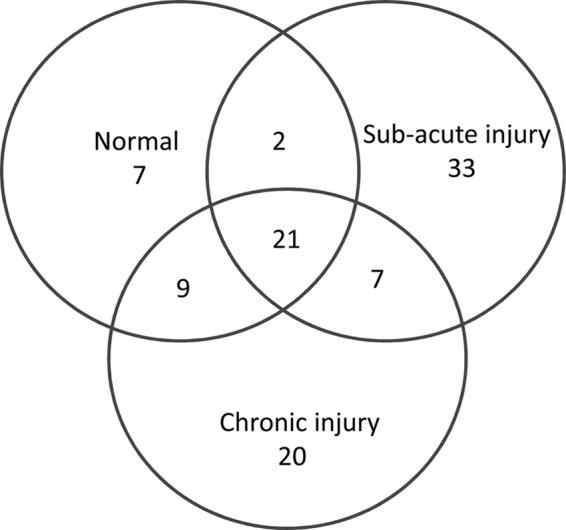
**Venn representation of proteins identified from Q-TOF LC-MS.** Samples of extracts of normal, sub-acute, and chronic injured equine SDFT samples (*n* = 3 for each) were analyzed for global protein composition, demonstrating differences in tendon ECM protein expression profiles with injury stage. Although a similar number of proteins was identified in both sub-acute and chronic injury phases, it was in the sub-acute phase injury that the greatest numbers of unique proteins were present.

Analyses of normal and natural diseased flexor tendons identified differences in protein expression profiles for a large number of proteins as summarized in Fig. 6. Twenty-one proteins were common to normal, sub-acute, and chronic injured tendons, although a greater number of additional proteins were identified in injured samples, including albumin, Tenascin-C, fibronectin, and annexin A1, A2, and A5. COMP was identified in sub-acute and chronic injured tendons but was not the most abundant ECM protein in contrast to normal samples. COMP neo-terminal fragments detected in extracts of normal, sub-acute, and chronic injured SDFTs are shown in Table 3. Four COMP neo-terminal fragments were identified in tissue extract samples of sub-acutely injured tendons and one in the chronic injury stage. Of these five COMP neo-terminal fragments identified in natural injury, C↓AVGWAGNGLLCGR↓D and F↓CFSQENIIWANLR↓Y were identified in the *in vitro* model system after stimulation with IL-1β. Quantitative MRM analyses showed that levels of the COMP neo-N-terminal fragment CFSQENIIWANLR, common to natural injury and the *in vitro* model, were elevated in media from IL-1β-stimulated tendon explants compared with non-stimulated controls and PGE_2_-stimulated samples.

**TABLE 3:** **Summary of COMP peptides unique to natural SDFT injury** Numbers indicate the peptide position within the equine COMP protein sequence.

Normal SDFT	Sub-acute SDFT injury	Chronic SDFT injury
228–238 C↓PDGTPSPCHEK↓A	254–266 C↓AVGWAGNGLLCGR↓D*^a^*	682–692 R↓WFLQHRPQVGY↓I
726–736 F↓SQENIIWANLR↓Y	652–663 A↓LWHTGDTASQVR↓L*^a^*	
	653–663 L↓WHTGDTASQVR↓L*^a^*	
	682–692 R↓WFLQHRPQVGY↓I	
	724–736 F↓CFSQENIIWANLR↓Y*^b^*	

*^a^* Peptides common to sub-acute tendon injuries and IL-1β−stimulated tendon explants.

*^b^* Levels of the F↓CFSQENIIWANLR↓Y peptide were increased in media from IL-1β stimulated tendon explants compared to controls.

## DISCUSSION

Stimulation of tendon explants with two proinflammatory mediators did not induce significant collagen release between 24 and 96 h. Hence, tendon explants exhibit similar behavior to cartilage with respect to the lack of collagens released during the early stages of culture (46, 47). However, it was not possible to maintain good cell viability in tendon explants beyond 5 days, which is why this time interval was selected as maximal for this study. The increased release of COMP with IL-1β supports a catabolic role of IL-1β in tendon similar to that reported for cartilage (28, 39, 40), which was further confirmed by the abrogating effects of IL-1Ra. Firocoxib but not Ilomastat inhibited IL-1β-induced COMP release. Previous studies have shown that proteinases other than matrix metalloproteinases may be responsible for COMP degradation *in vitro* by aggrecanases such as ADAMTS-4 (28), which are not inhibited by Ilomastat, although the effects of aggrecanase inhibitors were not explored in the current study.

Although we did not investigate the effects of strain on tendon, stress deprivation has been shown to induce IL-1 production (48), and stress deprivation may occur in injury, which may explain some of the neo-terminal peptides observed in diseased tendon. COMP was readily released from the matrix, which may be either the consequence of weaker interactions with matrix components compared with other matrix proteins or that the released COMP is newly synthesized. However, the presence of cleaved forms of COMP in control samples would be more consistent with a proteolytic-mediated release. Although cell death may have released intracellular proteases, it is unlikely that this was the major source of fragments because our control samples differentiated those fragments generated or substantially elevated after cytokine addition. IL-1β stimulation enhanced the release of cleaved forms of COMP and further degradation of monomeric COMP (<100 kDa, Fig. 3*B*). Neither the low nor high dose of PGE_2_ enhanced additional fragmentation patterns over control samples. However, MRM analyses showed trends for combined stimulation with IL-1β and PGE_2_ that produced differing effects depending on the concentration of PGE_2_. Stimulation with IL-1β and low dose PGE_2_ resulted in increased release of cleaved peptides of ECM proteins, whereas IL-1β and high dose PGE_2_ limited the number of neo-terminal cleavage sites. The synergy between low dose PGE_2_ and IL-1β on peptide release in this study is curious and could be explained by a number of hypotheses. First, the kinetics of prostaglandin receptor occupancy may be prolonged by the higher concentration of PGE_2_, leading to receptor desensitization, which would dampen receptor effects. Second, the presence of higher levels of PGE_2_ may exert an auto-regulatory feedback effect on IL-1 activity to modulate the inflammatory reaction (49). Third, the higher doses of PGE_2_ can activate resolution of inflammation in tendon fibroblasts via the production of specialized pro-resolving mediators such as lipoxins (50). This has been reported in an identical experimental system whereby the addition of 1.0 μm PGE_2_ to normal tendon explants induced maximal lipoxin A_4_ release after 72 h in tissue culture (3). We have demonstrated combined stimulation of explants with IL-1β and the same concentrations of PGE_2_ similarly induced lipoxin A_4_ release, with greater production with the higher dose of PGE_2_, suggesting that PGE_2_ may exert anti-catabolic effects on tendon ECM (36).

Comparative proteomic analysis of normal and naturally diseased tendons identified differences in protein/peptide profiles. The presence of annexin A1 identified only in the sub-acute and chronic injury samples implicate that both inflammatory and pro-apoptotic mechanisms are active (51) and continue into the later stages of tendon healing. COMP was also identified in samples of injured SDFT, but it was not the most abundant protein, in contrast to normal tendons, suggesting a change in the tendon protein profile after injury. Both injury phases had a similar number of proteins identified by proteomic analysis, although the greater number of proteins unique to sub-acute disease suggests that a large change in the phenotype of tendon cells is a feature of early disease stages, and this change to a large extent persists into chronic disease. This failure of the protein profile to return to normal suggests that injury permanently changes the composition of tendon ECM, which may compromise both the mechanical properties of the tissue and its influence on the responses of tenocytes in maintaining homeostasis. This may be a contributing factor to the high risk of re-injury in horses due to the formation of a repair scar with inferior mechanical properties to normal tendons (23, 52).

MRM analyses showed that levels of the F↓CFSQENIIWANLR↓Y COMP peptide in sub-acute injury were also elevated after IL-1β stimulation of tendon explants. MRM analyses suggest that the relative abundance of the F↓CFSQENIIWANLR↓Y fragment is greater after stimulation with IL-1 β rather than PGE_2_. Furthermore, the C↓AVGWAGNGLLCGR↓D COMP fragment was only identified after stimulation with IL-1β and not PGE_2_ (low or high dose), and therefore, the presence of these fragments *in vivo* provides supportive evidence that IL-1 is active in naturally occurring tendon injury.

The identification of novel COMP peptide cleavage sites common to both natural disease and an *in vitro* model of tendon inflammation provide a platform for the development of antibodies to identify the stage of tendon injury and enzyme inhibitors for therapeutic intervention. The combination of these disease-specific fragments may allow a multiplex marker platform to be developed for tendon injury.

## Footnotes

2 The abbreviations used are:

ECM: extracellular matrix
PG: prostaglandin
SDFT: superficial digital flexor tendon
Q-TOF: quadruple TOF
MRM: multiple reaction monitoring
COMP: cartilage oligomeric matrix protein
MMP: matrix metalloproteinase.
